# Functional analysis of drug resistance‐associated mutations in the *T* *rypanosoma brucei* adenosine transporter 1 (TbAT1) and the proposal of a structural model for the protein

**DOI:** 10.1111/mmi.12979

**Published:** 2015-03-21

**Authors:** Jane C. Munday, Daniel N. A. Tagoe, Anthonius A. Eze, Jessica A. M. Krezdorn, Karla E. Rojas López, Abdulsalam A. M. Alkhaldi, Fiona McDonald, Jennifer Still, Khalid J. Alzahrani, Luca Settimo, Harry P. De Koning

**Affiliations:** ^1^ Institute of Infection, Immunity and InflammationCollege of Medical, Veterinary and Life SciencesUniversity of GlasgowGlasgowG12 8TAUK; ^2^ Wellcome Trust Centre for Molecular ParasitologyUniversity of GlasgowGlasgowUK; ^3^ Department of Laboratory TechnologyUniversity of Cape CoastCape CoastGhana; ^4^ Department of Medical BiochemistryCollege of MedicineUniversity of NigeriaEnugu CampusEnuguNigeria; ^5^ Department of BiologyCollege of ScienceAljouf UniversitySakakaSaudi Arabia; ^6^ Faculty of Medical SciencesTaif UniversityTaifSaudi Arabia; ^7^ Department of Chemistry and Chemical Biology417 Egan Research CenterNortheastern University360 Huntington AvenueBostonMA02115USA

## Abstract

The *T* *rypanosoma brucei* aminopurine transporter P2/TbAT1 has long been implicated in the transport of, and resistance to, the diamidine and melaminophenyl arsenical classes of drugs that form the backbone of the pharmacopoeia against African trypanosomiasis. Genetic alterations including deletions and single nucleotide polymorphisms (SNPs) have been observed in numerous strains and clinical isolates. Here, we systematically investigate each reported mutation and assess their effects on transporter function after expression in a *tbat1* ^−/−^ *T* *. brucei* line. Out of a set of six reported SNPs from a reported ‘resistance allele’, none significantly impaired sensitivity to pentamidine, diminazene or melarsoprol, relative to the *TbAT* *1*‐WT allele, although several combinations, and the deletion of the codon for residue F316, resulted in highly significant impairment. These combinations of SNPs, and ΔF316, also strongly impaired the uptake of [^3^ H]‐adenosine and [^3^ H]‐diminazene, identical to the *tbat1* ^−/−^ control. The TbAT1 protein model predicted that residues F19, D140 and F316 interact with the substrate of the transporter. Mutation of D140 to alanine resulted in an inactive transporter, whereas the mutation F19A produced a transporter with a slightly increased affinity for [^3^ H]‐diminazene but reduced the uptake rate. The results presented here validate earlier hypotheses of drug binding motifs for TbAT1.

## Introduction

African trypanosomiasis is a disease complex covering a number of severe human and veterinary conditions, caused by *Trypanosoma brucei gambiense* and *T. b. rhodesiense* (human), and *T. b. brucei*, *T. congolense* and *T. vivax* (veterinary) respectively. Despite the recent introduction of nifurtimox‐eflornithine combination therapy (NECT) for late‐stage *gambiense* sleeping sickness (Alirol *et al*., 2013), melaminophenyl arsenicals (melarsoprol and cymelarsan) and diamidines (pentamidine and diminazene) continue to be used for all the African trypanosomiases (Holmes *et al*., 2004; Delespaux and de Koning, 2007; Kennedy, 2013). Because these drugs have been used for many decades, it is hardly surprising that resistance to most of these drugs has become very problematic (Barrett *et al*., 2011; Baker *et al*., 2013). Rates of resistance of between 20% and 39% have been reported for melarsoprol treatment in a wide variety of foci (Legros *et al*., 1999; Brun *et al*., 2001; Moore and Richer, 2001; Stanghellini and Josenando, 2001; Robays *et al*., 2008; Mumba Ngoyi *et al*., 2010), and the ease of inducing eflornithine resistance in a lab‐setting (Vincent *et al*., 2010) necessitated the identification of a partner drug to enable combination therapy, resulting in NECT treatment (Priotto *et al*., 2009). Drug resistance in African trypanosomes has been strongly linked to alterations in various transporters, including the amino acid transporter TbAAT6 for eflornithine (Vincent *et al*., 2010), P2/TbAT1 for arsenicals and diminazene (Carter *et al*., 1999; de Koning *et al*., 2004) and the High Affinity Pentamidine Transporter (HAPT1/AQP2) for arsenicals and pentamidine (de Koning, 2001a; Bridges *et al*., 2007; Munday *et al*., 2014).

The aminopurine transporter P2 was first linked to arsenical resistance when it was observed that this transport activity had disappeared from a *T. b. brucei* strain adapted to sodium melarsen and that adenine and adenosine could abrogate the effects of melaminophenyl arsenicals on trypanosomes (Carter and Fairlamb, 1993). Cloning of the encoding gene, designated *TbAT1*, allowed the identification of an allele from a melarsenoxide cysteamine‐resistant *T. b. brucei* clone that contained a number of nucleotide differences relative to the drug‐sensitive paternal strain, resulting in six amino acid changes: L71V, A178T, G181E, D239G, N286S and L380P. Unlike the wild‐type allele found in the sensitive strain, this allele failed to sensitise yeast cells to melarsen oxide (Mäser *et al*., 1999), and deletion of *TbAT1* in *T. brucei* caused reduced sensitivity to melaminophenyl arsenicals and pentamidine, and a high level of resistance to diminazene (Matovu *et al*., 2003). Moreover, these drugs were shown to bind with high affinity to the P2 transporter (de Koning and Jarvis, 1999) and to conform to the P2‐transporter binding motif (de Koning, 2001b).

In a series of clinical *T. b. gambiense* isolates, mostly from a focus in north‐western Uganda with a high incidence of melarsoprol treatment failure, 38 of 65 isolates contained a *TbAT1* allele that contained the same set of mutations, except L380P, as well as a codon deletion for residue F316, correlating significantly with relapse after melarsoprol treatment (Matovu *et al*., 2001). However, it has never been reported whether the full set of single nucleotide polymorphisms (SNPs) is required for resistance, or whether in fact only one of these might be responsible for the phenotype. This is important as a restriction‐length polymorphism and an allele‐specific polymerase chain reaction (PCR) have been proposed as diagnostic tools for the detection of melarsoprol resistance (Mäser *et al*., 1999; Matovu *et al*., 2001; Nerima *et al*., 2007). Furthermore, the mechanisms by which each of these SNPs might cause resistance through changes in the rate of TbAT1‐mediated drug transport have remained un‐investigated. Here, we systematically introduce each coding mutation that has been reported in a *TbAT1* ‘resistance allele’ and investigate their effects on the ability of the gene to affect drug sensitivity and drug transport in a *tbat1* ^−/−^ strain of *T. brucei*.

A TbAT1 protein model was created in order to predict the location and the effects of the mutations mentioned above in the transporter. In addition, the protein model was used to suggest new amino acid residues predicted to interact directly with the transported ligands. Two residues, F19 and D140, were identified in the binding pocket and were mutated, separately and in combination, to alanine residues, causing highly significant loss of diminazene transport and providing support for the proposed TbAT1 protein structure.

## Results and discussion

### Expression of mutant TbAT1 alleles in *T* *. brucei* strain B48

The *TbAT1* mutant vectors were constructed by site directed mutagenesis of pHDK44 (Munday *et al*., 2013), the expression plasmid containing the control Wild‐type (WT) TbAT1 allele, and further by rearrangement of the single mutation alleles to generate multiple mutation alleles within one expression plasmid. They were verified by Sanger sequencing, prior to transfection into the *T. brucei* strain B48 (Bridges *et al*., 2007). Clones produced following transfection were screened by PCR for the presence of the TbAT1 gene and to check that the plasmids were linear and had integrated correctly prior to use for drug resistance/uptake assays (data not shown).

### Analysis of single nucleotide polymorphisms in reported TbAT1 resistance alleles

It was previously shown that *TbAT1*, when expressed in yeast, sensitises these cells to melarsen oxide (Mäser *et al*., 1999). This gene, cloned from the melarsen‐sensitive *T. b. brucei* strain STIB777S, was also shown to mediate transport of the diamidines diminazene and pentamidine in yeast (de Koning *et al*., 2004). An alternative sequence, *TbAT1^r^*, obtained from the same strain adapted to melarsen oxide cysteamine (Cymelarsan^®^), STIB777R, did not sensitise yeast to melaminophenyl arsenicals. The resistance‐associated allele contained six coding SNPs, leading to the following mutations relative to the sensitive reference gene: L71V, A178T, G181E, D239G, N268S and L380P. The first five of these SNPs were then also reported in isolates from multiple relapse patients from north‐western Uganda unsuccessfully treated with melarsoprol (Matovu *et al*., 2001). In addition, these isolates contained a further mutation, namely the deletion of the codon for Phenylalanine 316. The same set of mutations was also found in a melarsoprol‐refractory *T. b. rhodesiense* isolate from Southern Uganda, and in a *T. b. gambiense* isolate from a melarsoprol relapse patient from Angola (Matovu *et al*., 2001). In all, several studies showed a clear association of the mutant alleles with melarsoprol resistance, although the correlation was not absolute (Mäser *et al*., 2001; Matovu *et al*., 2001; Kazibwe *et al*., 2009). However, it has never been reported which, or which combination, of the observed mutations was responsible for the apparent impairment of drug transport.

**Figure 1:**
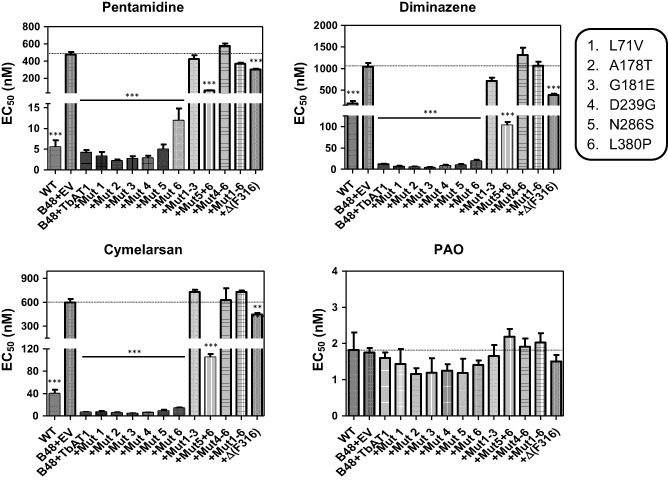
Effective concentration 50% (EC_50_) values for four trypanocidal drugs as determined using the Alamar Blue fluorescence assay. The strains tested were all derived from *T* *. b. brucei* s427 wild‐type (WT); B48 is a multidrug‐resistant strain lacking the genes for the TbAT1 and TbAQP2 drug transporters (Munday *et al*., 2014). B48 + EV is the resistant control of B48 transfected with the empty vector (EV) pHD1336 (Biebinger *et al*., 1997). All the variants of TbAT1 including the wild‐type copy (B48 + TbAT1) were expressed in B48 using this vector (Munday *et al*., 2013). Mutations (Mut) 1–6 are as listed in the box; Δ(F316) is the *TbAT* *1* allele from which the codon for Phe316 was deleted. The dotted line running across each panel denotes the EC50 value of the B48 + EV control for each drug. Statistical significance was determined using a one‐way ANOVA relative to B48 + EV, in GraphPad Prism 5.0. **, *P* < 0.01; ***, *P* < 0.001. Data shown are the average and SEM of at least five independent determinations.

We have therefore introduced each of the six STIB777R mutations and the F316 deletion separately in the reference sequence and expressed the mutant alleles in the multi‐drug resistance *T. b. brucei* line B48, which was derived from the tbat1 knockout strain tbat1^−/−^ (Matovu *et al*., 2003; Bridges *et al*., 2007) and assessed whether this affected the susceptibility phenotype to melaminophenyl arsenicals (cymelarsan) and diamidines (pentamidine, diminazene aceturate); phenylarsine oxide (PAO) was used as a control trypanocide that does not depend on the presence of the TbAT1 transporter (Carter and Fairlamb, 1993; Bridges *et al*., 2007). The EC_50_ values obtained for all four drugs were compared with those for the same cell line transfected with an ‘empty vector’ without insert (B48 + EV; drug‐resistant control) as well as with the same cells transfected with the wild‐type allele of *TbAT1* (B48 + TbAT1; drug‐sensitive control) and the parental strain Lister 427 (WT). As shown in Fig. 1, all of the individual mutation alleles displayed at least partial uptake activity for the cymelarsan and the diamidines, leading to highly significant susceptibility to these compounds, but not to PAO, relative to B48 + EV [*P* < 0.001; one‐way analysis of variance (ANOVA)].

None of the STIB777R SNPs identified by Mäser *et al*. (1999) significantly changed susceptibility to the test drugs compared with expression of the wild‐type TbAT1 transporter (B48 + TbAT1), although expression of TbAT1^L380P^ tended to increase the susceptibility of the B48 cells slightly less than the wild‐type allele (Fig. 1). The deletion mutation Δ(F316) identified in clinical isolates, however, appeared to almost completely abolish drug transport activity: its expression increased drug susceptibility only slightly, relative to B48 + EV, although still significantly. However, the line expressing the Δ(F316) allele was significantly more resistant to the test drugs, except PAO, than B48 + TbAT1 (*P* < 0.001).

As none of the STIB777R mutations individually appeared to give rise to a significant loss of drug transport activity, we investigated whether combinations of these SNPs together explain the lack of drug susceptibility reported by the STIB777R resistance allele (Mäser *et al*., 1999). Expression of a TbAT1 allele with mutations 5 (N286S) and 6 (L380P) together created a trypanosome with an intermediate susceptibility phenotype that was significantly more resistant than B48 + TbAT1 but significantly more sensitive than B40 + EV (*P* < 0.001; Fig. 1). Expression of alleles bearing mutations 1–3 (L71V / A178T / G181E), 4–6 (D239G / N286S / L380P) or all six mutations were completely resistant to diamidines and cymelarsan.

### Transport of adenosine and diminazene by TbAT1 transporters with STIB777R mutations

In order to verify that the drug susceptibility phenotypes described above are indeed linked to similar changes in transporter activity, we measured transport rates for 0.1 μM [^3^H]‐adenosine and for 0.1 μM [^3^H]‐diminazene. Adenosine uptake was conducted in the presence of 1 mM inosine in order to block transport through the P1‐type nucleoside transporters, as described (de Koning and Jarvis, 1999; Al‐Salabi *et al*., 2007).

**Figure 2:**
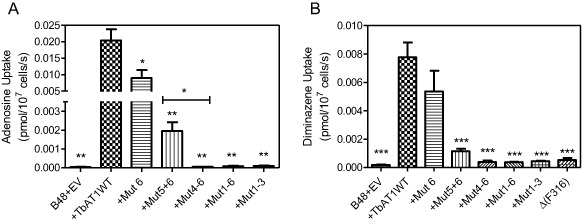
Transport of (A) 0.1 μM [^3^ H]‐adenosine or (B) 0.1 μM [^3^ H]‐diminazene aceturate by *T* *. b. brucei* B48 transfected with empty vector pHD1336 (B48 + EV) expressing either the wild‐type TbAT1 allele or a mutant TbAT1 allele as listed in Fig. 1. Incubation times were 30 s or 60 s for adenosine or diminazene transport respectively. All bars are the average of three independent experiments and error bars represent SEM; statistical significance was calculated relative to TbAT1WT (unless specifically indicated) using a paired Student's *t*‐test: *, *P* < 0.05; **, *P* < 0.02; ***, *P* < 0.01.

The mutations had virtually identical effects on transport of adenosine and diminazene (Fig. 2), suggesting that the general functioning of the transporter was affected rather than its specificity changed to disable the transport of diamidines while retaining its physiological function. Mutation 6 (L380P) alone caused only a slight decrease in adenosine and diminazene transport rates, which was significant for adenosine (*P* < 0.05; paired *t*‐test). Transport rates were further reduced in the mut5 + 6 double mutant (*P* < 0.01) but still remained above the level of the Mut4‐6 triple mutant (Fig. 2), indicating that the activity was not completely abrogated. For alleles with mutations 1–3, 4–6 and 1–6, transport of either substrate was barely detectable and not significantly different from the B48 + EV control while highly significantly different from B48 + TbAT1 (*P* < 0.01, paired *t*‐test; *n* = 3). Similarly, diminazene uptake in Δ(F316) was barely detectable (Fig. 2B). Thus, the transport results match the drug sensitisation profiles very well.

**Figure 3:**
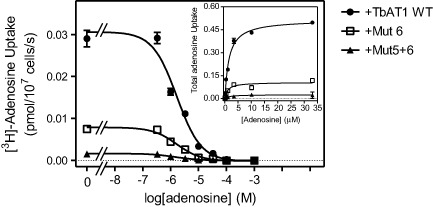
Transport of 0.1 μM [^3^ H]‐adenosine by B48 cells expressing various TbAT1 alleles as indicated (numbering as in Fig. 1), in the presence or absence of various concentrations of unlabelled adenosine. Main figure: experiment performed in triplicate with unlabelled adenosine as inhibitor, resulting in sigmoid competition curves. Inset: conversion of the same experiment to Michaelis‐Menten saturation plots displaying total adenosine uptake against adenosine concentration. The experiment depicted is representative of four independent experiments, which are summarised in Table 1.

We next investigated whether the transport rates were reduced because of reduced substrate affinity (K_m_), or because of impaired transport efficiency, expressed as V_max_/K_m_. To this end, transport of 0.1 μM [^3^H]‐adenosine was measured in the presence of various concentrations of unlabelled adenosine (Fig. 3), using B48 clones expressing either TbAT‐WT, TbAT1 mutation 6 alone or mutations 5 and 6. No attempt to obtain kinetic parameters for the other strains (+Mut4‐6, +Mut1‐6, +Mut1‐3) was undertaken as the TbAT1 transport activity could not be detected in these clones (Fig. 2). It was clear that no change had occurred in the substrate affinity, as the dose‐dependency of inhibition with unlabelled adenosine was unchanged (Fig. 3). However, the rate of uptake was again observed to be substantially lower in the mutant lines, and the conversion of the data to Michaelis‐Menten plots (Fig. 3, inset), clearly shows that it is the V_max_ rather than the K_m_ that is affected by the mutations; this could be either the result of reduced translocation rates by the mutant transporters, or due to reduced numbers of the mutant transporters in the plasma membrane. Table 1 gives the average values from four independent experiments, showing significant differences of V_max_ but not K_m_ values for both mutants.

**Table 1:** Effects of mutations in *TbAT* *1* amino acids 286 and 380 on kinetic parameters of adenosine transport

	TbAT1‐WT	TbAT1^L380P^	TbAT1^N286S+L380P^
K_m_ (μM)	0.82 ± 0.25	0.69 ± 0.13	1.02 ± 0.29
V_max_ (pmol(10^7^ cells)^−1^ s^−1^)	0.35 ± 0.07	0.12 ± 0.01^a^	0.027 ± 0.008^b^
V_max_/K_m_	0.43	0.17	0.026

V_max_ and K_m_ values for TbAT1‐mediated [^3^H]‐adenosine transport were determined in the presence of 1 mM inosine, using 0.1 μM radiolabel and 10 s incubations to determine the initial rates of transport. The data shown are the average and standard error of the mean of four independent experiments, each performed in triplicate, in which the parameters for all three strains was determined in parallel, using the same solutions and conditions.

a *P* < 0.02.

b *P* < 0.01 (unpaired Student's *t*‐test, relative to the wild‐type allele of TbAT1).

### Modelling the mutants

**Figure 4:**
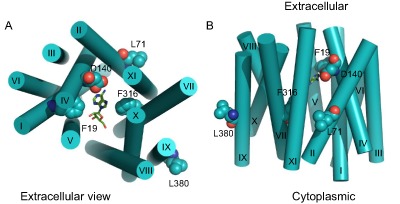
TbAT1 model created using ROBETTA. Residues whose mutation is described in this study are displayed in space‐filling models. The ligand adenosine is shown in green carbon atoms as docked in the TbAT1 protein model. Extracellular and intracellular intra TM‐helical loops were not modelled and are therefore not shown. Helices are indicated by rigid cylinders. Both top view (A; extracellular side) and side view (B) are shown. TM‐domains are labelled with roman numerals. The image was created using PyMol version 1.50.04, Schrödinger.

In order to rationalise the effect of the mutations described above, a protein model of the TbAT1 transporter was constructed using ROBETTA as described in the method section. The top‐ranked model (model 1) produced by ROBETTA was structurally aligned with the LdNT1.1 model published by Valdes *et al*. (2009; 2012). This structural alignment is shown in Fig. S1 in the supplementary information section. It is interesting to note that the two protein model structures can be easily structural aligned despite the fact they were created using different methodologies and have dissimilar protein sequences. In fact Valdes *et al*. built the LdNT1.1 model using structure fragments generated by the standard ROSETTA fragment server in combination with a secondary structure prediction method (Valdes *et al*., 2009); the TbAT1 protein model described in the present study was instead created using partial threaded protein models using two different crystal structure templates. This seems to confirm that, despite the limited sequence similarity (amino acid sequence 29% identity; 50% similarity), these two structurally related proteins are expected to recognise and transport adenosine in a similar way, in agreement with the observation that, in evolution, structure is more conserved than sequence (Holm and Sander, 1996). Figure 4 displays the positions of those residues positioned within the transmembrane (TM)‐helices that were mutated in the present study. The mutations L71V, L380P and the deletion of F316 have been putatively linked in the literature to melarsoprol resistance (Mäser *et al*., 1999) but were never mapped in a TbAT1 protein model. The model suggests that F316 is in close proximity with the ligand, whereas L71 and L380 are located far from the substrate translocation channel (Fig. 4). A deletion of F316 is expected to have an important effect on the binding and transport of the substrate – not just because it is expected to contribute to substrate binding but also because the deletion changes the position and orientation of the subsequent amino acid residues in the TM‐helix. The experimental results seem in agreement with this prediction showing that the F316 deletion is substantially impaired in the uptake of different substrates (Figs 1 and 2). Clearly, the mutation L380P is predicted to have a detrimental effect for the folding of the TM‐helix because the proline residue cannot donate an intra‐helical amide hydrogen bond (having no amide hydrogen), and also because its side‐chain interferes sterically with the backbone of the same helix (Richardson, 1981). The mutants A178T, G181E, D239G, N286S also published in the literature (Mäser *et al*., 1999) are located in intracellular or extracellular loops that are notoriously difficult to model and are not displayed in Fig. 4 so it is difficult to understand the effect of these mutations using molecular modelling techniques.

**Figure 5:**
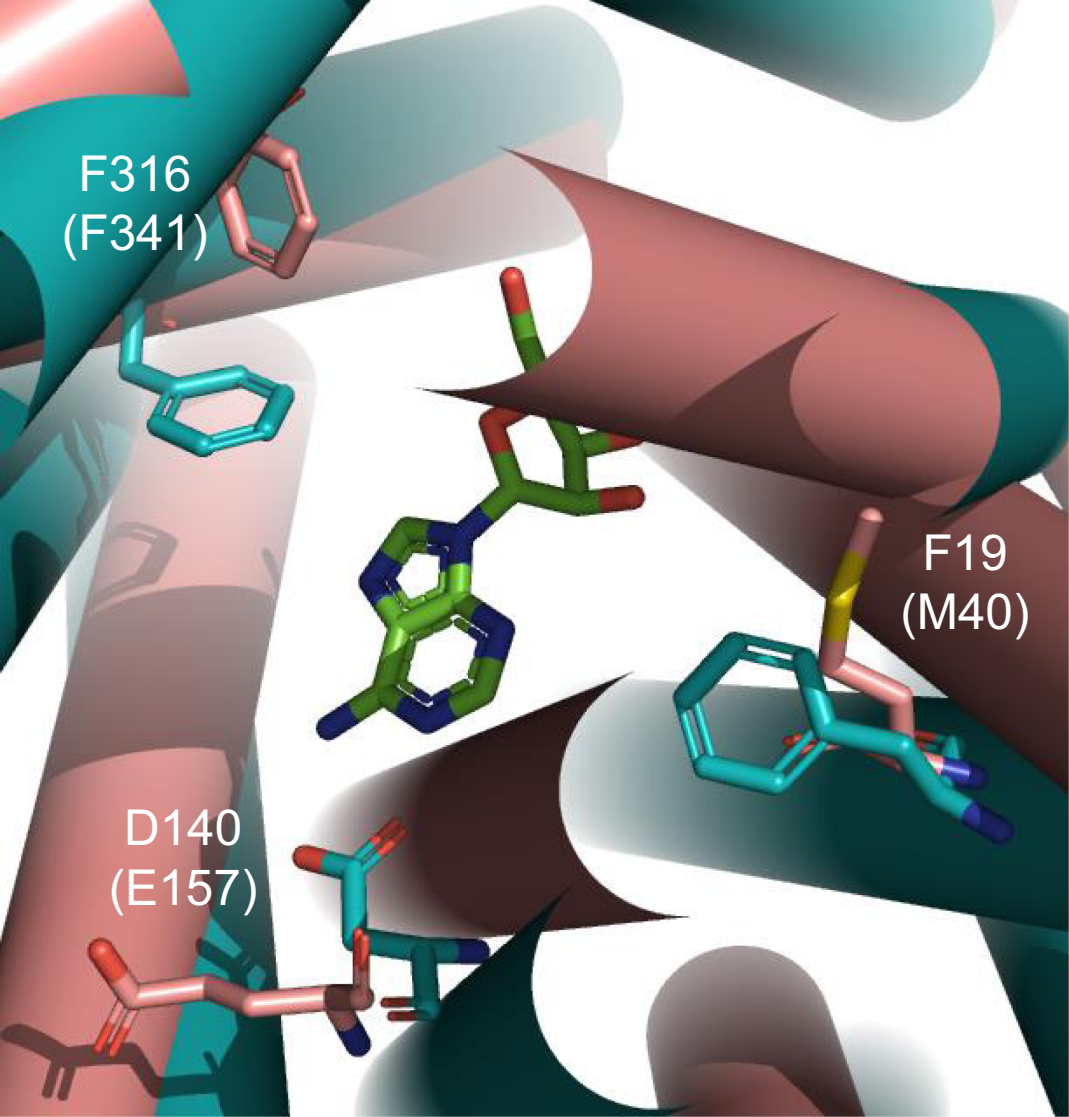
Structural overlay between the LdNT1.1 model published by Valdes *et al*. (2009; 2012) – in brown; and the TbAT1 model obtained created by ROBETTA – in cyan. The extracellular side is shown. The ligand adenosine docked in the TbAT1 protein model is shown in green carbon atoms. Helices are indicated by rigid cylinders. The extracellular and intracellular TM‐helical loops were not modelled and are therefore not shown. The TbAT1 residue numbering is used, with the LdNT1.1 residues in brackets. The image was created using PyMol version 1.50.04, Schrödinger.

**Figure 6:**
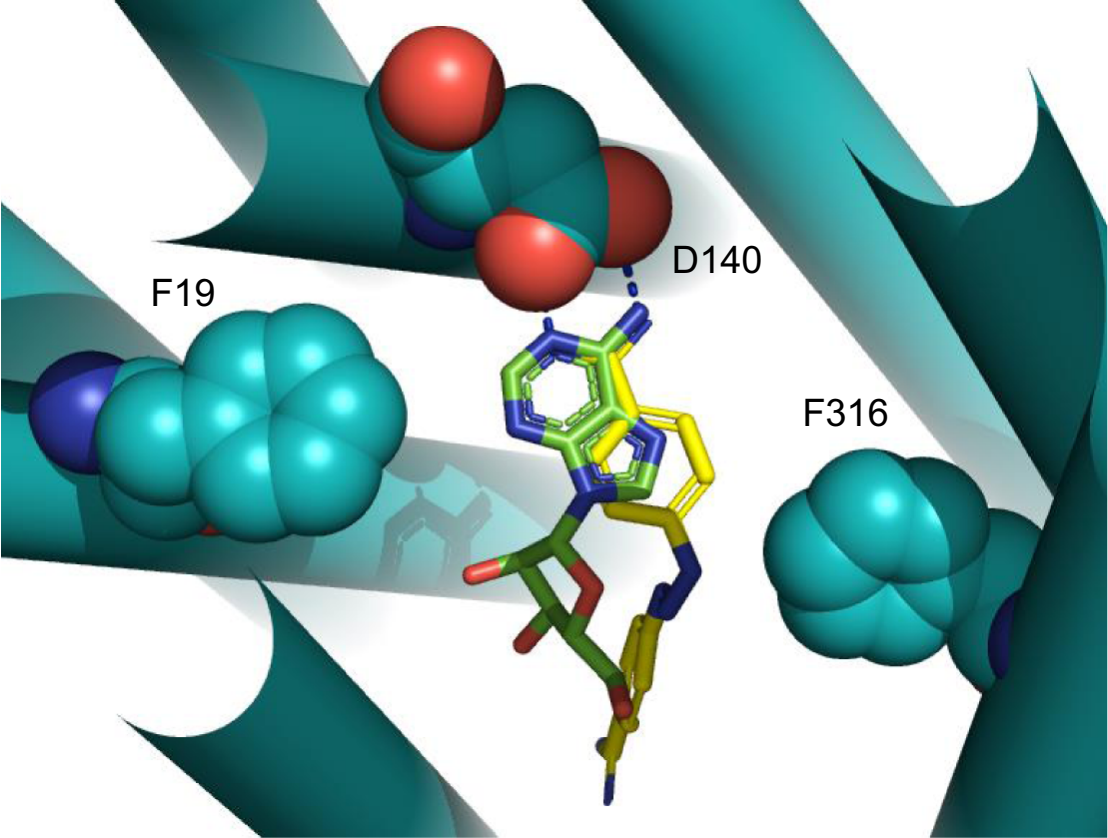
Overlay between the predicted binding poses of adenosine (green carbon atoms) and diminazene (yellow carbon atoms) in the TbAT1 model obtained created by ROBETTA (colored in cyan). Residues F19, D140 and F316 are displayed in space‐filling models. Helices are indicated by rigid cylinders. The hydrogen bonds between the side‐chain carboxylate group of Asp140 and (i) the amino group and (ii) the protonated nitrogen‐1 of the adenosine ring are shown in dashed blue lines. The image was created using PyMol version 1.50.04, Schrödinger.

**Figure 7:**
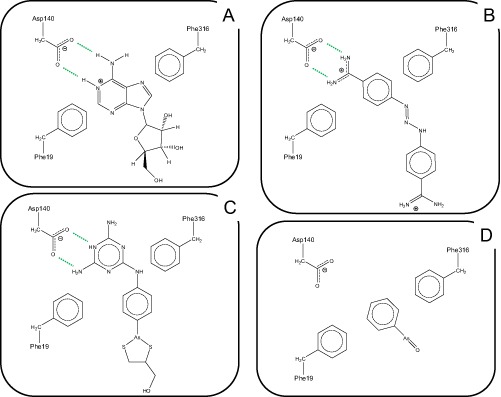
Schematic model for the interactions between TbAT1 ligands adenosine (A), diminazene (B) and melarsoprol (C) with the critical amino acid residues in the TbAT1 binding pocket; Frame (D) shows the putative interaction of phenylarsine oxide with the binding pocket. Hydrogen bonds are depicted as dotted lines. The image was created using ChemDraw Pro 10.0, CambridgeSoft.

Two new mutations were introduced in order to test the predictions of the protein model with respect to ligand binding: F19A and D140A (Figs 4, 5, 6). The structural alignment of TbAT1 and LdNT1 (Fig. 5) suggested a hypothesis for the residues involved in the recognition and transport of adenosine. The knowledge of the key residues affecting substrate specificity and/or adenosine uptake activity in LdNT1.1 (Valdes *et al*., 2009) helped to study *in silico* the protein–substrate interactions in TbAT1. F316, F19 and D140 are in proximity to the docked adenosine in a pocket located within the channel, near the extracellular side (Figs 4, 5, 6). These residues are structurally aligned with F341, M40 and E157 (in LdNT1.1) respectively (Fig. 5). Mutations of each of these three residues in LdNT1.1 are known to affect substrate specificity or result in a loss of adenosine uptake activity (Valdes *et al*., 2009) suggesting that these residues play a key role in the binding and transport of the substrate. In particular, the side‐chain of D140 of TbAT1 is predicted to give a bidentate interaction with the ligand accepting two hydrogen bonds: from (i) the amino group of adenosine and (ii) nitrogen‐1 of the purine ring (Fig. 6). The nitrogen‐1 in the adenine ring is in fact protonated at physiological pH as shown experimentally (Christensen *et al*., 1970; Zimmer and Biltonen, 1972; Major *et al*., 2002) and using quantum‐mechanical calculations (Turecek and Chen, 2005). Valdes *et al*. reported that the mutation E157 to aspartate or glutamine caused a change in substrate specificity or a loss of more than 95% of adenosine uptake respectively (Valdes *et al*., 2009). Our study suggests that D140 in TbAT1 (and therefore also E157 in LdNT1.1) is predicted to accept hydrogen bonds from the purine ring (Fig. 6). D140 is predicted to give a similar bidentate interaction with the melamine moiety of cymelarsan or the amidine group of pentamidine and diminazene (Figs 6 and 7A–C). Interestingly, phenylarsine oxide does not contain any hydrogen bond donor functionality, suggesting there would be no interaction with D140 (Fig. 7D), which is consistent with the previously reported TbAT1‐independent uptake of this compound (Carter and Fairlamb, 1993; Bridges *et al*., 2007). F19 in TbAT1 is instead structurally aligned with M40 in LdNT1.1. Both phenylalanine and methionine are hydrophobic residues, and it has been reported that the latter residue is also capable of π‐interaction similarly to aromatic residues (Tatko and Waters, 2004). The proposed binding mode of adenosine to TbAT1 presented in this study is in agreement with previous studies published in the literature that suggest the presence of: (i) hydrogen‐bonds with the nitrogen‐1 and the amino‐group of the adenine ring; and (ii) π‐stacking with the adenine aromatic system (de Koning and Jarvis, 1999; de Koning *et al*., 2005; Lüscher *et al*., 2007). Our model suggests that the two hydrogen bonds mentioned above are accepted by the carboxylate group of D140, whereas the π‐stacking could be given by F19 and/or F316 that are predicted to be located in proximity of the ligand.

### Drug sensitivity profile of predicted binding pocket mutants

**Figure 8:**
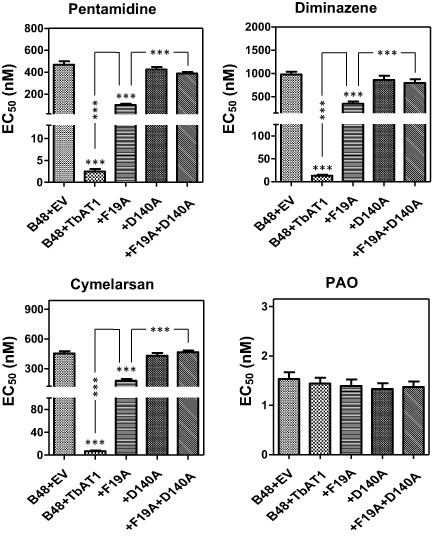
EC_50_ values obtained by the Alamar Blue test for strains expressing mutant alleles of TbAT1. Bars represent the average and SEM of six independent experiments. Statistical significance was determined by a one‐way ANOVA, compared to B48 + EV except where specifically indicated.

The susceptibility of these mutants to diamidines and arsenicals is displayed in Fig. 8. The mutant alleles F19A and D140A had little effect on the susceptibility to pentamidine, diminazene and cymelarsan, relative to the B48 + EV line, whereas the cells expressing the wild‐type TbAT1 allele were sensitised to all; PAO had an identical effect on all cells lines. The F19A mutant was somewhat more susceptible than the empty vector control (*P* < 0.001; one‐way ANOVA), as well as much less susceptible than the TbAT1‐WT control (*P* < 0.001). The D140A mutation, however, appeared to produce a completely inactive transporter, as susceptibility of the B48 + EV and B48 + TbAT1^D140A^ cell lines to the test drugs was not significantly different. Combining both mutations resulted in an equally inactive transporter.

### Transport of diminazene by TbAT1^F19A^

**Figure 9:**
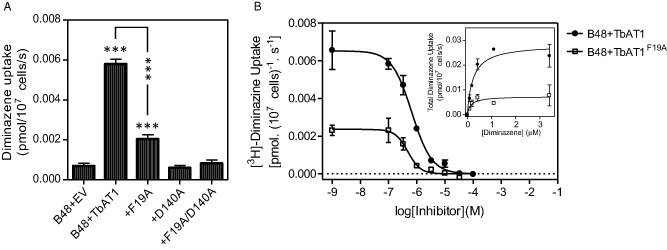
Uptake of 0.1 μM [^3^ H]‐diminazene is significantly reduced following reintroduction of mutated TbAT1 relative to the wild type TbAT1. A. Transport of diminazene was assessed in each strain. Bars show mean rate of transport ± SEM at 1 min when normalised for background, *n* = 4. * = *P* < 0.05, ** = *P* < 0.01, *** = *P* < 0.001 (one‐way ANOVA and Dunnett's test), compared against B48 + EV, unless otherwise indicated. B. Inhibition (main figure) and saturation plot (inset) of 0.1 μM [^3^ H]‐diminazene over 60 s in the presence or absence of various concentrations of unlabelled diminazene as indicated. The experiment show was performed in triplicate and representative of four independent experiments, which are summarised in Table 2.

The rate of 0.1 μM [^3^H]‐diminazene transport was significantly reduced in cells expressing the TbAT1^F19A^, TbAT1^D140A^ and TbAT1^F19A+D140A^ mutations, when compared with cells expressing TbAT1‐WT (all *P* < 0.001, *n* = 4). Of the three mutant lines, only diminazene uptake in the B48 + TbAT1^F19A^ line was significantly faster than B48 + EV (*P* < 0.01; Fig. 9A); there was no significant difference in diminazene uptake by B48 + EV and B48 + TbAT1^D140A^ or B48 + TbAT1^F19A+D140A^. As with the STIB777R‐derived mutants that still displayed quantifiable transport rates, we determined the K_m_ and V_max_ values for the F19A mutant (Fig. 9B). Surprisingly both parameters were significantly lower compared with the wild‐type TbAT1 allele (Table 2). Although this results in a transporter with a threefold higher affinity for diminazene, the actual rate of transport was much reduced, possibly because of restrictions in the required conformational change, or the proton symport on which the *T. brucei* purine transporters are dependent (de Koning and Jarvis, 1997a, 1997b; de Koning *et al*., 1998); additionally, it is expected that the stronger binding by the mutant transporter might result in a reduced off‐rate for the release of the substrate into the cell. However, the overall rate of transport by the mutant transporter is dependent on both the translocation rate of each transport protein (catalytic activity) and the number of such proteins in the plasma membrane, either or both of which might be affected by the mutation. In summary, our results show that F19, D140 and F316 (Fig. 6) are key residues in the protein channel responsible for the interaction with substrates and this is in agreement with the model proposed.

**Table 2:** Effects of the F19A mutation on kinetic parameters of diminazene transport

	TbAT1‐WT	TbAT1^F19A^
K_m_ (μM)	0.64 ± 0.15	0.21 ± 0.03^a^
V_max_ (pmol(10^7^ cells)^−1^ s^−1^)	0.040 ± 0.006	0.007 ± 0.002^b^
V_max_/K_m_	0.062	0.034

V_max_ and K_m_ values for TbAT1‐mediated [^3^H]‐diminazene transport was determined using 0.1 μM radiolabel and 60 s incubations to determine the initial rates of transport. The data shown are the average and standard error of the mean of four independent experiments, each performed in triplicate, in which the parameters for both strains was determined in parallel, using the same solutions and conditions.

a *P* < 0.02.

b *P* < 0.01 (unpaired Student's *t*‐test, relative to the wild‐type allele of TbAT1).

The results presented here provide new insights into the molecular causes of drug resistance in African trypanosomes of the *brucei* group. Remarkably, no individual SNPs previously reported in resistance‐associated *TbAT1* alleles had a significant effect on drug sensitivity in *T. brucei*, as the introduction of a *TbAT1* allele with any one of those SNPs sensitised a *tbat1* ^−/−^ strain in the same way as the wild‐type *TbAT1* allele. Only the mutation L380P, which was not present in the resistance alleles isolated from the melarsoprol‐relapse patients in Uganda and Angola (Matovu *et al*., 2001), appeared to affect drug sensitivity although this did not reach statistical significance. However, the combination of two or three, or all six, of these mutations did render the drug transport activity mostly (N286S/L380P), or completely, defective. Importantly, the single mutation ΔF316 also resulted in an allele that confers partial resistance to diamidines and cymelarsan and was unable to transport [^3^H]‐diminazene.

The results are thus in complete agreement with the reports that associate this set of mutations with drug resistance and melarsoprol treatment failure (Mäser *et al*., 1999; Matovu *et al*., 2001), although we must emphasise that (i) not all mutations in these alleles are required in order to have complete loss of drug transport through *TbAT1* and (ii) other mutations may similarly cause loss of TbAT1 function. For instance, we have previously reported on three laboratory‐adapted MPXR strains (one each of *T. b. brucei*, *T. b. gambiense* and *T. equiperdum*) that did not introduce any of the *TbAT1* mutations here described but either lost the complete *TbAT1* open reading frame or *TbAT1* expression (Stewart *et al*., 2010). Similarly, the *T. b. rhodesiense* strain STIB900 had lost the *TbAT1* ORF after *in vitro* adaptation to melarsoprol (Bernhard *et al*., 2007), and a new mutant *TbAT1* was found after adaptation to pentamidine (Munday *et al*., 2014). Yet, the presence of an ‘TbAT1 resistance allele’ identical to that reported by Matovu *et al*. (2001), including the same set of silent mutations, was recently confirmed in two MPXR *T. b. brucei* isolates from Somalia and an MPXR *T. b. rhodesiense* isolate from Uganda (Graf *et al*., 2013); the only other *TbAT1* mutation in a field isolate was a tri‐nucleotide deletion in two isolates from the Democratic Republic of Congo, *TbAT1*‐Δ516–518, which introduces one SNP and deletes an amino acid from the fifth predicted TM domain (Pyana Pati *et al*., 2014). We conclude that the mutations here investigated are stable in trypanosome populations in Eastern Africa and highly relevant to drug resistance in the treatment of livestock with diminazene and of late‐stage rhodesiense sleeping sickness with melarsoprol (the only treatment available (Delespaux and de Koning, 2007)).

A protein model of TbAT1 was built and then structurally aligned with the LdNT1.1 protein model validated by mutagenesis by Valdes *et al*. (2009). This suggests that four of the SNPs (A178T, G181E, D239G and N286S) are located in extracellular or intracellular loops and that the other two (L71V and L380P) are located in TM domains remote from the predicted binding site. Thus, the likely rationale for the observed phenotypes is that none of the SNPs is directly involved in ligand binding and that it takes several such mutations to affect the structure to the point of destroying the drug transport activity. The reason that L380P had an effect on the transport, when compared with the other single SNP mutants, is most likely due to the introduction of a proline residue (a residue known to destabilise α‐helices) in the middle of a TM‐helix domain.

Visual inspection showed that the most favourable energy‐minimised pose of diminazene displayed a strong bidentate interaction between the guanidine group of this ligand and the carboxylate group of Asp140 (a residue known to also play a key role in substrate recognition in LdNT1.1 (Valdes *et al*., 2009). These are well‐known, strong hydrogen‐bond interactions, whose energetics have been investigated in detail using experimental methods (Linton and Hamilton, 1999). Interestingly, the latter study shows that the bidentate interaction between a guanidine group and a carboxylate is associated with an experimental enthalpic change of −3.6 kcal mol^−1^ (−15.1 KJ mol^−1^). Collar *et al*. (2009) show, based on ligand binding studies, that the amino group and the nitrogen atom 1 of adenosine (the atoms that we predict to be involved in this bidentate interaction) is predicted to contribute for a total of 7.7 + 8.2 = 15.9 KJ mol^−1^ to the interactions of adenosine with TbAT1, which is remarkably similar to the experimental enthalpic contribution of a bidentate H‐bond such as with Asp140.

When we introduced mutations in any of the residues that our model predicted to be directly involved in ligand binding (F19, D140 and F316), the result was a highly significant or a complete loss of drug transport ability. Particularly, the mutation of aspartate 140 to alanine resulted in a completely non‐functional transporter with respect to drug sensitivity and diminazene transport. This is not surprising as our previously published results (de Koning and Jarvis, 1999; Collar *et al*., 2009) clearly identified a –N = C‐NH_2_ motif as essential for ligand binding by TbAT1. This motif corresponds to the protonated purinic nitrogen N1 and the amine group in position 6 of adenosine, the amidine group of pentamidine and other diamidines, such as diminazene and DB75, and the melamine ring of melarsoprol and cymelarsan (de Koning *et al*., 2005). Our model suggests that these hydrogen bonds are established with the side‐chain carboxylate group of aspartate residue at position 140. The previous studies of the P2 ligand‐binding motif also predicted an important contribution from π‐orbital interactions between the purine ring (or benzamidine, or melaminophenyl moieties) and aromatic residues in the binding pocket, which we here identify as F19 and F316.

We conclude that the TbAT1 protein model described here is consistent with the functional analysis presented in the current manuscript and with the extensive ligand binding and transport studies conducted on this important determinant of trypanosomal drug sensitivity and resistance.

## Experimental procedures

### Trypanosome strains and culturing

Bloodstream‐form *T. b. brucei* strain B48 and its derivatives were maintained as previously described (Teka *et al*., 2011). The B48 strain is derived from the Lister 427 strain, but lacks the *T. brucei TbAT‐1* gene and lost the high affinity pentamidine transport activity (Bridges *et al*., 2007) as a result of a genomic rearrangement in the *TbAQP2‐AQP3* locus (Baker *et al*., 2012).

### Plasmid construction and transfection

All six mutations in the *T. brucei* P2 aminopurine transporter (TbAT1) identified by Matovu *et al*. (2001) were introduced into pHDK44 (Munday *et al*., 2013), which was based on pHD1336 (Biebinger *et al*., 1997), by site directed mutagenesis, using the QuikChange II kit (Agilent, Santa Clara, CA, USA), following the manufacturer's instructions.

The following primers were used to generate TbAT1 mutations: for L71V, primers HDK347 & 348, producing plasmid pHDK51; for L380P, primers HDK349 & 350, plasmid pHDK52; for A178T, primers HDK351 & 352, giving plasmid pHDK53; for G181E, primers HDK353 & 354, giving plasmid pHDK54; for D239G, primers HDK355 & 356, to produce pHDK55 and for the mutation N286S, primers HDK357 & 358, giving plasmid pHDK56 (all primer details given in Supplementary Table S1).

Combinations of these individual mutations were constructed by either further site directed mutagenesis or by digestion and ligation of relevant fragments from the individual plasmids. The plasmid containing *TbAT1* with both the N286S and L380P mutations was made by digestion of the two single mutation plasmids using *Sac*II. The dropout from pHDK55 (N286S) was ligated into the backbone of pHDK56 (L380P), to give plasmid pHDK58. Following this, the further mutation, D239G, was inserted into plasmid pHDK58 by site directed mutagenesis, using primers HDK353 & 354, to give plasmid pHDK59. Plasmid pHDK57, containing mutations A178T and G181E, was generated by site directed mutagenesis of plasmid pHD52, using primers HDK359 & 360. The mutation L71V was further added to give plasmid pHDK61, by digestion of pHDK51 and pHDK57 with *Mfe*I and *Not*I. Finally, all six mutations were combined into one plasmid by digestion and re‐combination of pHDK59 and pHDK61 using *Bsp*MI and *Alw*NI, to give plasmid pHDK62.

The deletion of codon F316 (also from Matovu *et al*., 2001) was completed by site directed mutagenesis of pHDK44 using the primers HDK469 & 470, giving plasmid pHDK64. The two active site mutations were also introduced by site directed mutagenesis, using primers HDK483 & 484 for mutation F19A, giving plasmid pHDK65 and the primers HDK485 & 486 for D140A, to produce plasmid pHDK66.

All plasmids were checked by Sanger Sequencing (Source BioScience, Nottingham, UK) for the presence of the correct mutation(s) and linearised with *Not*I prior to transfection. B48 parasites were washed into Human T Cell Solution for transfection of the desired cassette with an Amaxa Nucleofector as described (Burkard *et al*., 2007). Transfectants were grown and cloned out, by limiting dilution, in standard HMI‐11 (Hirumi and Hirumi, 1989) containing 5 μg ml^−1^ blasticidin S. Correct integration of the expression cassettes was analysed by PCR.

### Drug sensitivity assays

Drug sensitivities were assessed using the dye resazurin (Alamar blue) using a protocol adapted from Räz *et al*. (1997), as described (Gould *et al*., 2008; 2013). Briefly, drugs were serially diluted in 100 μl of complete HMI‐11 media across two rows of a 96 well plate. Cultures of bloodstream‐form trypanosomes were diluted in complete HMI‐11, and 100 μl was added to all wells. Plates were incubated for 48 h at 37°C/5% CO_2_, prior to the addition of 20 μl of 5 mM resazurin sodium salt (Sigma‐Aldrich) in PBS, pH7.4. Plates were incubated for a further 24 h in the same conditions, before fluorescence was measured using a FLUOstar Optima fluorimeter (BMG Labtech). The 50% effective concentrations (EC_50_) were calculated using the equation for a sigmoidal curve with variable slope of Prism 5 (GraphPad). Experiments were performed independently at least five times.

### Transport assays

Uptake assays of [^3^H]‐diminazene and [^3^H]‐adenosine were performed exactly as described (Wallace *et al*., 2002; Natto *et al*., 2005). Briefly, trypanosomes from late‐log phase cultures were washed into assay buffer (AB; 33 mM HEPES, 98 mM NaCl, 4.6 mM KCl, 0.55 mM CaCl_2_, 0.07 mM MgSO_4_, 5.8 mM NaH_2_PO_4_, 0.3 mM MgCl_2_, 23 mM NaHCO_3_, 14 mM glucose, pH 7.3) and resuspended at 10^8^ cells ml^−1^. One hundred microlitres of cell suspension was incubated with either [Ring‐^3^H]‐diminazene aceturate (Perkin Elmer, 60.7 Ci mmol^−1^), or [2,8‐^3^H]‐adenosine (American Radiolabeled Chemicals Inc, 40 Ci mmol^−1^), in the presence or absence of unlabeled substrate or other competitive inhibitors. Incubations were stopped by centrifugation through oil (13 000× *g*; 1 min). The cell pellet was transferred to a scintillation tube, and radioactivity was determined by liquid scintillation counting. The results were plotted to equations for linear or non‐linear regression using Prism 5 (GraphPad) after correction for non‐specific association of radiolabel with the pellet, as described (Wallace *et al*., 2002).

### Creation of the protein model and docking

The protein sequence of the TbAT1 was obtained from direct Sanger sequencing of the gene in Lister 427 parasites. Five different protein models of TbAT1 were created using ROBETTA (http://www.robetta.bakerlab.org). This full‐chain protein structure prediction server allows protein models to be built via homology modelling and/or *ab initio* modelling using Rosetta (Kim *et al*., 2004; Raman *et al*., 2009; Song *et al*., 2013). The derived protein models were structurally aligned with the *Leishmania donovani* Nucleoside Transporter 1.1 (LdNT1.1) model built using Rosetta *ab initio* modelling software published by Valdes *et al*. (2009; 2012). The authors of these studies validated their model using site‐directed mutagenesis and kindly provided us with the co‐ordinates of their protein model. The TbAT1 model that gave the best structural alignment with LdNT1.1 model was the top‐ranked model (model 1) suggested by ROBETTA and was chosen to study the protein–ligand interactions *in silico* described in this study. In order to create this protein model, the Ginzu Domain Prediction in ROBETTA identified two domains of the TbAT1 that were modelled separately: (i) domain 1–267 with some similarity to the structure of a bacterial fucose transporter [PDB code: 3O7Q; (Dang *et al*., 2010)] and (ii) domain 268–463 with some similarity with a bacterial glycerol‐3‐phosphate transporter [PDB code: 1PW4 (Huang *et al*., 2003)]. These two partial threaded protein models were assembled together as a last step in ROBETTA. Molecular docking was performed using FRED (McGann, 2012), a docking programme that uses an exhaustive search algorithm and was used to dock molecules generated from a multiconformer database generated using OMEGA. The docked poses were energy minimised using SZYBKI (v 1.7.0 OpenEye Scientific Software, Santa Fe, NM, USA) allowing partial relaxation of the protein residues in the direct proximity to the ligand. FRED, OMEGA and SZYBKI are software developed by OPENEYE (OpenEye Scientific Software: Santa Fe, NM, USA).

## Supporting information

Supporting information
