# Supplementary material for: Functional analysis of drug resistance‐associated mutations in the T rypanosoma brucei adenosine transporter 1 (TbAT1) and the proposal of a structural model for the protein
Source: Mol Microbiol. 2015 Mar 21;96(4):887–900. doi: 10.1111/mmi.12979 (PMC4755147; doi:10.1111/mmi.12979)
Supplement: Supplementary file 1 — Supporting information [file MMI-96-887-s001.zip › mmi_12979_SM.docx]

**SUPPLEMENTAL MATERIAL**

A protein model of the Trypanosoma brucei Adenosine Transporter 1 (TbAT1) validated by site-directed mutagenesis

Jane C. Munday, Daniel N. A. Tagoe, Anthonius A. Eze, Jessica A. M. Krezdorn, Karla E. Rojas López, Abdulsalam A. M. Alkhaldi, Fiona McDonald, Jennifer Still, Khalid J. Alzahrani, Luca Settimo and Harry P. De Koning

Legend to Figure S1.

Figure S1. Structural overlay between the LdNT1.1 model published by Valdes and co-workers (Valdés, Arastu-Kapur, Landfear, & Shinde, 2009; Valdés, Shinde, & Landfear, 2012) - in brown - and the TbAT1 model obtained created by Robetta - in cyan. The ligand adenosine (bound and transported by both proteins) is shown in green carbon atoms as docked in the TbAT1 protein model. Helices are indicated by rigid cylinders. Both top view (A) and side view (B) are shown. Extracellular and intracellular intra TM-helical loops were not modeled and are therefore not shown. The image was created using PyMol version 1.50.04, Schrödinger.
