# Supplementary figures and images for: Functional analysis of drug resistance‐associated mutations in the T rypanosoma brucei adenosine transporter 1 (TbAT1) and the proposal of a structural model for the protein
Source: Mol Microbiol. 2015 Mar 21;96(4):887–900. doi: 10.1111/mmi.12979 (PMC4755147; doi:10.1111/mmi.12979)

A

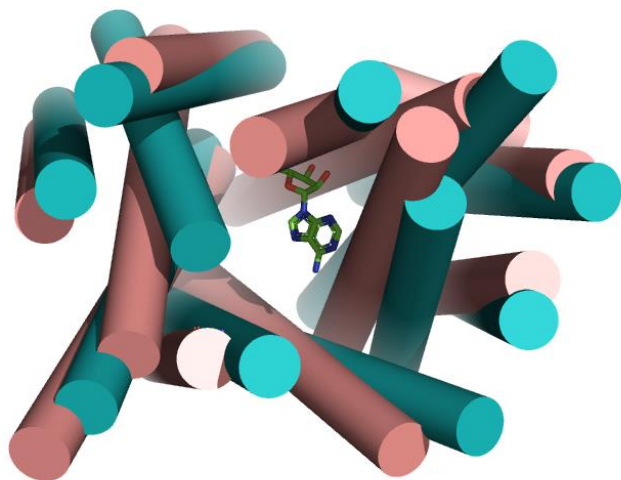

Extracellular view

B

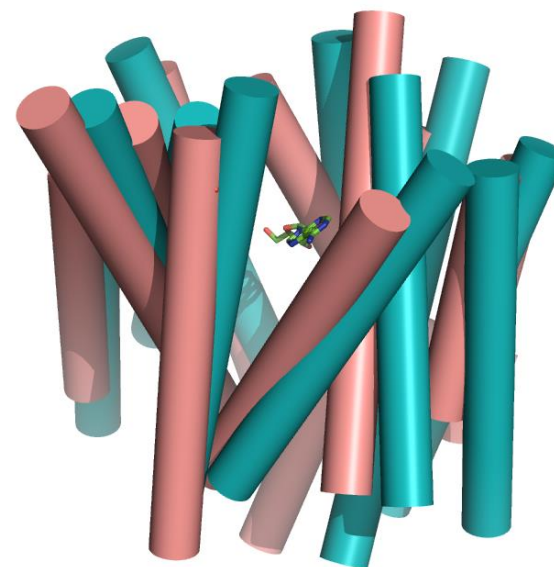

Cytoplasmic

Supplement: Supplementary file 1 — Supporting information [file MMI-96-887-s001.zip › mmi_12979_FS1.pdf]
