# Supplementary material for: Functional analysis of drug resistance‐associated mutations in the T rypanosoma brucei adenosine transporter 1 (TbAT1) and the proposal of a structural model for the protein
Source: Mol Microbiol. 2015 Mar 21;96(4):887–900. doi: 10.1111/mmi.12979 (PMC4755147; doi:10.1111/mmi.12979)
Supplement: Supplementary file 1 — Supporting information [file MMI-96-887-s001.zip › mmi_12979_Supp_TS1.doc]

**Supplementary Table S1: primers used for insertion of mutations into TbAT1**

| Primer | Mutation | Sequence (altered base(s) underlined) |
| --- | --- | --- |
| HDK347 | L71V | CTACAGTATTGCAGCGTTCGTCGTAGAGTTGGTTTTGGC |
| HDK348 | L71V | GCCAAAACCAACTCTACGACGAACGCTGCAATACTGTAG |
| HDK349 | A178T | CTTCTCGATCGTCATAAAAACATCCATGGGAGGCGGTTA |
| HDK350 | A178T | TAACCGCCTCCCATGGATGTTTTTATGACGATCGAGAAG |
| HDK351 | G181E | GTCATAAAAGCATCCATGGAAGGCGGTTATCACAACATG |
| HDK352 | G181E | CATGTTGTGATAACCGCCTTCCATGGATGCTTTTATGAC |
| HDK353 | D239G | ATTGATGATAAGGGCGCAGGTGGTGACGAAGGAAACGGC |
| HDK354 | D239G | GCCGTTTCCTTCGTCACCACCTGCGCCCTTATCATCAAT |
| HDK355 | N286S | GACATGGACCAGGTGGAAAGCATCACGACTTCGCAGCAG |
| HDK356 | N286S | CTGCTGCGAAGTCGTGATGCTTTCCACCTGGTCCATGTC |
| HDK357 | L380P | ACATTTGCGCGTATGCTGCCCATTATCCCACTTGTGCTT |
| HDK358 | L380P | AAGCACAAGTGGGATAATGGGCAGCATACGCGCAAATGT |
| HDK359 | A178T & G181E | GTCATAAAAACATCCATGGAAGGCGGTTATCACAACATG |
| HDK360 | A178T & G181E | CATGTTGTGATAACCGCCTTCCATGGATGTTTTTATGAC |
| HDK469 | ΔF316 | CTGTTCGCATGCTTCATGGTTTTCACCACATTTCTCGTCTAC |
| HDK470 | ΔF316 | GTAGACGAGAAATGTGGTGAAAACCATGAAGCATGCGAACAG |
| HDK483 | F19A | CTACGTCACCTTCCTCTTCGCCGGAATGTCGGTGGTGGTG |
| HDK484 | F19A | CACCACCACCGACATTCCGGCGAAGAGGAAGGTGACGTAG |
| HDK485 | D140A | GTAGCGATGACGCTTTGCGCTGCTGGAAACGCCGCACTC |
| HDK486 | D140A | GAGTGCGGCGTTTCCAGCAGCGCAAAGCGTCATCGCTAC |

**Supplementary figure legend:**

Figure S1. Structural overlay between the LdNT1.1 model published by Valdes and co-workers in brown, and the TbAT1 model created by ROBETTA in cyan. The ligand adenosine (bound and transported by both proteins) is shown in green carbon atoms as docked in the TbAT1 protein model. Helices are indicated by rigid cylinders. Both top view (A; extracellular side) and side view (B) are shown. Extracellular and intracellular intra TM-helical loops were not modeled and are therefore not shown. The image was created using PyMol version 1.50.04, Schrödinger.
